# Synergistic Plant-Microbe Interactions between Endophytic Actinobacteria and Their Role in Plant Growth Promotion and Biological Control of Cotton under Salt Stress

**DOI:** 10.3390/microorganisms10050867

**Published:** 2022-04-21

**Authors:** Osama Abdalla Abdelshafy Mohamad, Yong-Hong Liu, Li Li, Jin-Biao Ma, Yin Huang, Lei Gao, Bao-Zhu Fang, Shuang Wang, Ashraf F. El-Baz, Hong-Chen Jiang, Wen-Jun Li

**Affiliations:** 1 State Key Laboratory of Desert and Oasis Ecology, Xinjiang Institute of Ecology and Geography, Chinese Academy of Sciences, Urumqi 830011, China; liuyh@ms.xjb.ac.cn (Y.-H.L.); majinbiao@ms.xjb.ac.cn (J.-B.M.); yinhuang0929@163.com (Y.H.); gaolei19@mails.ucas.ac.cn (L.G.); fangbaozhu2009@126.com (B.-Z.F.); 2 Department of Biological, Marine Sciences and Environmental Agriculture, Institute for Post Graduate Environmental Studies, Arish University, Al-Arish 45511, Egypt; 3 Department of Environmental Protection, Faculty of Environmental Agricultural Sciences, Arish University, Al-Arish 45511, Egypt; 4 Heilongjiang Academy of Black Soil Conservation & Utilization, Heilongjiang Academy of Agricultural Sciences, Harbin 150086, China; wangshuang0726@163.com; 5 Department of Industrial Biotechnology, Genetic Engineering and Biotechnology Research Institute (GEBRI), University of Sadat City, Sadat City 32897, Egypt; ashraf.elbaz@gebri.usc.edu.eg; 6 State Key Laboratory of Biogeology and Environmental Geology, China University of Geosciences, Wuhan 430074, China; jiangh@cug.edu.cn; 7 State Key Laboratory of Biocontrol, Guangdong Provincial Key Laboratory of Plant Resources, School of Life Sciences, Sun Yat-sen University, Guangzhou 510275, China

**Keywords:** environmental microbiology, medicinal plants, endophytes, biofertilizer, biocontrol, *Verticillium dahliae*, actinobacteria, *Thymus roseus*

## Abstract

Bacterial endophytes are well-acknowledged inoculants to promote plant growth and enhance their resistance toward various pathogens and environmental stresses. In the present study, 71 endophytic strains associated with the medicinal plant *Thymus roseus* were screened for their plant growth promotion (PGP), and the applicability of potent strains as bioinoculant has been evaluated. Regarding PGP traits, the percentage of strains were positive for the siderophore production (84%), auxin synthesis (69%), diazotrophs (76%), phosphate solubilization (79%), and production of lytic enzymes (i.e., cellulase (64%), lipase (62%), protease (61%), chitinase (34%), and displayed antagonistic activity against *Verticillium dahliae* (74%) in vitro. The inoculation of strain XIEG05 and XIEG12 enhanced plant tolerance to salt stress significantly (*p* < 0.05) through the promotion of shoot, root development, and reduced the activities of antioxidant enzymes (SOD, POD, and CAT), compared with uninoculated controls in vivo. Furthermore, inoculation of strain XIEG57 was capable of reducing cotton disease incidence (DI) symptoms caused by *V. dahliae* at all tested salt concentrations. The GC-MS analysis showed that many compounds are known to have antimicrobial and antifungal activity. Our findings provide valuable information for applying strains XIEG05 and XIEG12 as bioinoculant fertilizers and biological control agent of cotton under saline soil conditions.

## 1. Introduction

On a global scale, salinization of soil is one of the most representative environmental stressors and is a major factor that decreases agricultural productivity and causes a loss of land area of about 2000 ha per day, contributing to 1–2% agricultural soil losses every year worldwide, resulting in decreased crop yield up to 70%, mainly in arid and semi-arid regions, causing a loss of US $27.3 billion per year [1,2,3]. Concisely, the problem of soil salinization is not only a scourge for agricultural productivity, but also affects soil physicochemical properties and the ecological balance of the area. Several studies have shown that salinity affects almost all factors of plant growth development, including physiological processes, causing nutrient deficiency, reduced vegetative growth, chlorophyll content, inhibited photosynthesis and protein synthesis, and increased susceptibility to phytopathogens [4,5,6,7]. Antioxidant enzymes are low molecular weight antioxidants produced by plants to confer salt stress tolerance. Remarkably, it has been estimated that the world population will exceed 9 billion people and more than 50% of arable land will be salt-affected due to global climate change by 2050 [8,9]. Undeniably, global climate change is a threat in the 21st century and is increasingly becoming a menace to all life on Earth. In contrast, and in response to salinity stress, arable land is declining gradually; hence, it is predicted that agricultural productivity cannot meet the requirements of an exploding world population, which would require an alternative strategy to crop improvement to enhance salt tolerance. Consequently, in recent years, researchers have been trying to investigate the available salinity management techniques to resolve the problem and provide several possible sustainable solutions for salinity reclamation and mitigation. 

Endophytic bacteria are ubiquitous in nature, residing inside all parts of plant tissues of most ecosystems and completing their life cycle, mostly in a facultative or obligate symbiotic relationship [10,11,12]. Endophytes are beneficial microorganisms that are important for the development of eco-friendly and sustainable agriculture practices since they help to improve crop yields by enhancing plant growth and protecting crops from microbial diseases [13,14,15]. In recent decades, endophytic actinobacteria have been shown to be attractive natural sources with great potential for the production of bioactive compounds for pharmaceutical and agricultural industries [16,17,18]. Furthermore, beneficial endophytic bacteria have been demonstrated to augment plant growth directly through nutrient solubilization, and the release of plant growth regulators or phytohormones such as indole acetic acid (IAA) enhance a symbiotic nitrogen fixation [19,20,21], as well as reduce infestation by phytopathogens through various mechanisms, including the production of hydrolyzing enzymes such as cellulases and chitinase. The production of siderophores cause degradation of fungal cell walls, aiding in lysis of hyphae and controlling spores germination [22,23,24], as well as the stimulation of systemic acquired resistance and tolerance to abiotic stress factors [25,26,27]. 

It has been well documented that medicinal plants have been traditionally used worldwide for the treatment of various diseases, and according to the World Health Organization (WHO), about 80% of the world population still relies on medicinal herbs due to their ability to produce a diverse array of biologically active compounds [28]. The composition of bioactive secondary metabolites synthesized by herbal medicines varies widely depending on the plant species [24,29,30]. Thymus is a traditional medicinal herb in the mint family (*Lamiaceae*) and is well known worldwide for its ability to synthesize high quantities of essential oils, which have been widely used in pharmaceutical and food applications [31,32,33,34]. Individually, beneficial endophytic actinobacteria living within various plant tissues of herbal medicines have the potential for the production of many secondary metabolites with application in agriculture and pharmaceutical industries [13,14,15,16,17,35]. Cotton (*Gossypium*) is one of the most important natural fiber crops and is used as an edible oil and biofuel worldwide. However, cotton faces several biotic and abiotic stresses; at present, about three million hectares of cotton in China are infected by the soilborne fungus *Verticillium dahliae*, accounting for an annual loss of 10–30% of the cotton yield [36,37]. Xinjiang province, located in the northwest of China, produces about 11% of the global cotton fiber and is disproportionately harmed by Verticillium wilt disease [38]. Nowadays, the screening of endophytic actinobacteria for their functional role is a promising eco-friendly and bio-economical approach for crop improvements and is gaining prominence [39,40]. Thus, the application of salt-tolerant plant growth-promoting (PGP) bacteria to salt- hypersensitive crops, such as maize, bean, tomato, pepper, and cotton, might increase agricultural production during salt-stress conditions [41,42,43,44,45]. In a first study of ongoing research, we previously reported the isolation and identification of endophytic actinobacteria associated with wild populations of the Chinese medicinal herb *Thymus roseus* Schipcz [46]. In recent decades, significant advances have been made in the endophytic bacteria interactions with plants. However, studies on the role of endophytic bacteria under stress conditions are still limited. Therefore, the principal goals of the present study were to (1) screen the beneficial endophytic actinobacterial strains associated with wild *T. roseus* ex situ; (2) evaluate their ability to stimulate growth-promoting and salt stress tolerance in cotton plants in vivo; (3) evaluate their biological control efficiency against *Verticillium* wilt disease in vitro/vivo; and (4) identify the major antimicrobial compounds produced by endophytes in the presence of *V. dahliae*, which are likely to be effectors of the antifungal properties. To the best of our knowledge, this is the first report of beneficial plant-microbe interactions associated with the wild medicinal plant *T. roseus* to evaluate their potential as biocontrol agents and PGPs in cotton in vitro/vivo.

## 2. Materials and Methods

### 2.1. In Vitro Screening for Plant Beneficial Traits and Antifungal Activities

#### 2.1.1. Determination of Nitrogenase Activity

In a first study of ongoing research, we previously isolated and characterized 71 endophytic actinobacterial strains associated with the wild medicinal plant *Thymus roseus* from the Tacheng site of Xinjiang, China under [46]. In this study, 71 strains previously isolated and characterized [46] were used to discover the effectiveness and potential application of beneficial actinobacteria and their role in plant growth promotion and biological control of cotton under salt stress in vitro and in vivo.

To test nitrogenase activity, we used two nitrogen-free media: Ashby’s mannitol agar media, composed of (L^−1^) (0.2 g MgSO_4_; 0.1 g CaSO_4_; 5.0 g CaCO_3_; 10.0 g mannitol; 0.2 g KH_2_PO_4_; 0.2 g NaCl; 15.0 g agar; pH 7.0) and NFC media (0.2 g MgSO_4_∙7H_2_O; 5.0 g CaCO_3_; 0.2 g KH_2_PO_4_; 10.0 g mannitol; 0.2 g NaCl; 0.2 g CaSO_4_∙2H_2_O; 15.0 g agar; at pH 7.2) [47,48]. All strains were inoculated separately on selective nitrogen-free media at 28 ± 1 °C for 8 days. The nitrogenase activity was observed based on the colony growth on Ashby’s and NFC agar plates. 

#### 2.1.2. Indole Acetic Acid (IAA) Production

Salkowski’s colorimetric method was used to determine the ability of endophytic strains associated with wild *T. roseus* to produce indole-3-acetic acid (IAA). The pure culture of each strain was grown in 25 mL of TYC broth (5 g L^−1^ tryptone; 0.872 g L^−1^ CaCl_l2_∙2H_2_O; and 3 g L^−1^ yeast extract) with 0.1% (*w*/*v*) L- tryptophan for 3–5 days at 28 ± 1 °C at 130 rpm [48,49]. After incubation, stationary-phase broth culture was centrifuged at 8000 rpm for 5 min, and the supernatant was mixed with 1 mL of Salkowski reagent (98 mL of 35% HClO_4_, and 2 mL of 0.5 M FeCl_3_) (1:1 *v*/*v*) and then kept in the dark at room temperature for 25–30 min. The formulation of pink color indicated indole production. Afterward, results were confirmed by measuring the optical density (OD_530_) nm in a 96-well microplate using a multimode reader and compared with known amounts of Indole Acetic Acid (IAA) using the Salkowski reagent and sterile TYC broth with tryptophan as blanks [47].

#### 2.1.3. Detection of Siderophores

Production of siderophores was observed based on competition for iron between ferric complexes of universal chrome azurol S/Fe (III)/hexadecyltrimethyl-ammonium bromide (CAS) agar media as described by [47]. All tested strains were incubated at 28 ± 1 °C for 6–8 days. Change of media color from blue to an orange/purple or red/purple halo zone around the colony was scored as positive for the production of siderophores [48,50].

#### 2.1.4. Determination of Phosphate Solubilization Ability

Testing the solubilization of inorganic phosphate activity was determined by using Pikovskaya’s agar medium supplemented with precipitated tricalcium phosphate Ca_3_(PO_4_)_2_ (5 g L^−1^) and Bromophenol Blue (0.025 g L^−1^), as described by [48,50], with some modifications. After eight days of incubation at 28 ± 1 °C, the development of yellow halos and/or clearing zones indicated the utilization of tricalcium phosphate present in the agar medium.

#### 2.1.5. Assays for Proteolytic, Lipolytic, Cellulolytic, and Chitinolytic Activity

Endophytic actinobacterial strains were checked for lipase enzyme activity by using the spot inoculation technique on modified Sierra lipolysis agar supplemented with ferrous citrate C_6_H_5_FeO_7_ (0.2 g L^−1^) and beef extract (3 g L^−1^). After sterilization, 50 mL of Victoria Blue B reagent solution (0.1 g per 150 mL) followed by 10 mL of Tween 80 was added to the Sierra lipolysis medium. After 7–8 days of incubation at 28 ± 1 °C, white calcium precipitates around colonies indicated a positive reaction [47].

Protease activity was assayed using the spot inoculation technique of endophytic actinobacterial strains on skim milk agar 5% (*v*/*v*) medium [51]. The skim milk agar plates were incubated for 5 days at 28 ± 1 °C. Protease activity was identified by the formation of a clear halo zone around colonies due to hydrolysis of skim milk.

Colloidal chitin medium was prepared from commercial chitin made from crab shells (C_8_H_13_NO_5_) n provided by Solarbio Life Science and followed the protocol of Agrawal and Kotasthane [52,53]. Chitinase detection medium consisted of (L^−1^) 3.0 g of (NH_4_)_2_SO_4_, 4.5 g of colloidal chitin, 0.3 g of MgSO_4_.7H_2_O, 2.0 g of KH_2_PO_4_, 1.0 g of citric acid monohydrate, 15 g of agar, 200 μL of Tween-80, and 0.15 g of bromocresol purple per liter, and then sterilized at 121 °C for 20 min. Endophytic actinobacteria chitinase activity was assessed by observation of colored zones around colonies.

Cellulase enzyme activity was tested using modified DSMZ medium 65 (http://www.dsmz.de/microorganisms/medium/pdf/DSMZ_Medium65.pdf (accessed on 16 March 2022)) without CaCO_3_ and supplemented with carboxymethyl cellulose (CMC) (5 g L^−1^; Sigma) in place of the carbon source glucose by using the spot inoculation technique of all tested endophytic actinobacterial strains. After incubation for 6–8 days at 28 ± 1 °C, plates were stained with a Congo red solution and then destained using a NaCl solution [53,54]. A clear or lightly colored halo zone around colonies indicated a positive reaction of cellulase enzyme activity.

#### 2.1.6. Screening for Antifungal Activities In Vitro

All 71 strains were screened for antagonistic activity in vitro against *Verticillium dahliae* Kleb using the plate diffusion method [55]. The fungal strain *V. dahliae* was grown in potato dextrose agar (PDA) plates for 8 days and a 5 mm agar plug was transplanted into the center of a 7 cm PDA plate. All tested strains were pre-grown individually in ISP_2_ media for 5 days. Bacterial strains were symmetrically spotted onto the four corners of a 7 cm plate, 2.5 cm from the plate periphery. All PDA plates were wrapped with parafilm and incubated at 25 ± 1 °C for 10 days and observed for the inhibition of *V. dahliae*. The antagonistic activity was quantified by measuring the inhibition zone of the pathogenic growth [56]. The inhibition percent was calculated using the following formula [53]:

$$\mathrm{Percent}\;\mathrm{inhibition}\;\left(\%\right)=\frac{F_{a}-T_{b}}{F_{a}-F_{0}}\;\times \;100$$

where (F_a_) is the fungal colony diameter on the control, T_b_ is the fungal colony diameter of the PDA treatment, and (F_0_) is the diameter of test fungus agar discs (approximately 5 mm). All screening experiments for plant beneficial traits in vitro were performed twice with three replicates for each strain.

### 2.2. In Vivo Salt Stress Experiment under Greenhouse Conditions

#### 2.2.1. Plant Growth-Promoting Activity in Saline Soil

The endophytic actinobacterial strains *Streptomyces luteus* (XIEG05), *Nocardiopsis dassonvillei* (XIEG12), and *Alloactinosynnema album* (XIEG20) were tested in vivo for stimulating effects of plant growth in saline soils. Upland cotton (*Gossypium hirsutum* “Yumian-1”) seeds provided by Manasi cotton research center. Cotton seeds were surface-sterilized by immersion in 20% (*v*/*v*) H_2_O_2_ for 2 min and then rinsed three times with ion-free distilled water [57]. Seeds were then germinated on wet filter paper in a Petri dish. The Petri dishes were wrapped with parafilm to avoid evaporation and placed in a plant growth chamber at 25 °C for 4 days. All tested strains were then inoculated in ISP_2_ broth media and incubated at 28 ± 1 °C for 4 days, and the cell suspension was centrifuged at 5000 rpm for 10 min. The cell pellets were resuspended at a final concentration of 10^8^ CFU/mL with phosphate-buffered saline PBS and adjusted using Densicheck plus (Biomerieux, Rodolphe, Durham, NC, USA) [48]. Cotton seedlings were inoculated by soaking roots for 10 min in a 5 mL solution of each endophytic actinobacterial strain suspension and gently agitated for a few minutes [58]. Cotton seedlings were aseptically transplanted into plastic pots (12 cm high × 10 cm in diameter) filled with sterilized low nutrition mixed soil composed of peat, compost, sand, and perlite (1:1:1:1, *v*/*v*) and placed in a greenhouse at 25 ± 1 °C with an average day/night period 14/8 h. After three days, 10 mL of bacterial suspension (10^8^ CFU/mL) was added near the root zone of each cotton plant. Salinity was increased gradually by applying a sodium chloride solution to each pot on alternative days to avoid osmotic shock to reach final salt concentrations of 50, 100, 150, and 200 mM, which was achieved after 3, 6, 9, and 12 days, respectively [48,59]. Parallel controls were maintained by cultivating cotton with sterilized compost without inoculation of endophytic actinobacterial strains and irrigating with tap water whenever needed. Each salt treatment contained three pots and each pot included four cotton seedlings. After 60 days, plants were harvested and analyzed for growth parameters and antioxidant enzyme assays. Plants were uprooted and washed to remove adhering peat. Shoot, root length, and fresh weight were recorded.

#### 2.2.2. Biological Control of *V. dahliae* under Salt Stress

Endophytic actinobacterial strain *Streptomyces albidoflavus* (XIEG24), *Curtobacterium flaccumfaciens* (XIEG29), and *Nocardiopsis alba* (XIEG57) showing preliminary antagonistic activity against *Verticillium dahliae* strain V991 in vitro were tested for their ability to control *V. dahliae* under salt stress in vivo. Cotton seedlings were aseptically planted in plastic pots filled with sterilized low-nutrition soil as described above. *V. dahliae* from 7-day-old potato dextrose broth (PDAB) was filtered with 4 layers of sterile gauze to remove mycelia, spores of *V. dahliae* were washed with sterile distilled water, and then diluted to a concentration of 10^8^ conidia/mL (Biomerieux, Rodolphe, Durham, NC, USA) [48,57]. Bacterial suspensions were prepared as described above. The treatments were: (i) control with *Verticillium dahliae* strain V991 only; and (ii) fungal pathogen with bacteria. Once seedlings presented with two true leaves, 10 mL of *V. dahliae* suspensions were poured into the soil surrounding the roots using a sterile syringe [53,57]. Then, three days after pouring *V. dahliae* suspensions, 10 mL of the endophytic actinobacterial suspension (10^8^ CFU/mL) was applied near the root zone as described above. Disease symptoms were recorded after 60 days following pathogen challenge. 

#### 2.2.3. Disease Assessment

Cotton disease severity was classified into four grades according to visible symptoms on cotyledons and true leaves as follows: 0 (no symptoms), 1 (>0–25% yellowing or wilting leaves), 2 (25–50% yellowing or wilting leaves), 3 (50–75% yellowing or wilting leaves), and 4 (75–100% yellowing or wilting leaves) [60]. The disease index (DI) (%) represents a comprehensive and objective measure of plant health, with high DI values corresponding to serious infection by *V. dahliae*. Disease index was calculated according to the following formula: DI = [(∑ disease grades × number of disease leaves)/(total number of leaves × 4)] × 100 [53].

### 2.3. Plant-Microbe Defense Response to Pathogens and Photosynthetic Pigments under Salt Stress

#### 2.3.1. Determination of Antioxidant Enzymatic Activity

Cotton leaf extracts were used to measure antioxidant enzymes as described by [61]. Cotton leaves were ground using liquid nitrogen and stored at −80 °C. The ground leaf samples (approximately ∼1 g) were homogenized on ice using 10 mL 50 mM phosphate buffer (pH 7.8) and then incubated for 10 min at 4 °C. Subsequently, the homogenate was filtered using Advantech Qualitative Filter Papers (110 mm) and centrifuged at 6000× *g* for 15 min at 4 °C. The supernatant was used for the determination of enzyme activities. The activities of superoxide dismutase (SOD), catalase (CAT), and peroxidase (POD) were measured using assay kits (kit Numbers. A001-1, A007-1, A003-3-1, and A084-3, respectively; Nanjing Jiancheng Bioengineering Institute, China), following the manufacturer’s instructions (http://elder.njjcbio.com/index_en.php, accessed on 20 December 2021) [48]. All experiments were performed on ice and conducted in triplicate.

#### 2.3.2. Photosynthetic Pigments

Chlorophyll content was quantified by using the Leaf Chlorophyll Meter (SPAD 502 Plus). Readings were taken on the uppermost fully expanded leaf with a visible collar during vegetative growth, and from the ear leaf (12 leaves per treatment) as suggested [48,62].

### 2.4. Extraction and Identification of Metabolites 

#### 2.4.1. Isolation and Purification of Bioactive Compounds

An antibiosis experiment was conducted by co-cultivation of strains *Streptomyces albidoflavus* (XIEG24), *Curtobacterium flaccumfaciens* (XIEG29), and *Nocardiopsis alba* (XIEG57) with *V. dahliae* in 500 mL^−1^ of broth medium at 28 °C for 20 days with agitation speed at 180 rpm in triplicate. Cells were collected by centrifugation at 6000 rpm for 15 min. The cell-free supernatant was mixed with an equal volume (1:1) of ethyl acetate by vigorous shaking for 60 min at room temperature and allowed to separate. The organic solvent phase was evaporated at 43 ± 1 °C under vacuum, using a rotary evaporator (IKA, HB10 basic). The ethyl acetate extract was dissolved in 5 mL of Tris-Cl buffer (0.02 M, pH 7.0) and analyzed using gas chromatography/mass spectrometry (GC-MS) [48,53].

#### 2.4.2. Identification of Bioactive Compounds

GC-MS analysis of the endophytic actinobacterial cell-free extracts was performed using a gas chromatograph (Model 7890A, Agilent, Palo Alto, CA, USA) equipped with a split-splitless injector, an Agilent model 7693 autosampler, and an Agilent HP-5MS fused silica column (5% Phenyl-methylpolysiloxane, 30 m length, 0.25 mm I.D., film thickness 0.25 mm). Injecting volume was 0.8 µL, and heating from 50 to 300 °C at 10 °C/min, followed by 10 min at 300 °C. The injector was maintained at 280 °C. Helium was used as the carrier gas, at 1.0 mL min^−1^, and the split mode was 5:1. The GC was fitted with a quadrupole mass spectrometer with an Agilent model 5975 detector. The MS conditions were as follows: ionization energy, 70 eV; electronic impact ion source temperature, 230 °C; quadrupole temperature, 150 °C; scan rate, 3.2 scans/s; mass range, 40–800 µ. The compounds were identified based on the match with their mass spectra and retention indices according to NIST/Wiley 275 library (Wiley, New York). The Relative abundance of each feature was calculated from the Total Ion Chromatogram (TIC) computationally [48]. 

### 2.5. Statistical Analysis

The data represent mean of values represent an average of 10–12 10–12 replicates ± standard error (SE) calculated by MS Excel. One-way ANOVA was used to compare the means of root length, root fresh weight, shoot length, shoot fresh weight, SOD, POD, CAT, and Chlorophyll content for each salt concentration separately, and Tukey’s HSD post-hoc test was used for multiple comparisons at α = 0.05. Statistical analyses were conducted in R (R Core Team). R Core Team (2018). R: A language and environment for statistical computing. R Foundation for Statistical Computing, Vienna, Austria. URL https://www.R-project.org/, accessed on 31 March 2020.

## 3. Results

### 3.1. In Vitro Screening of Endophytic Actinobacteria Strains for Plant Growth-Promoting Traits

**Figure 1:**
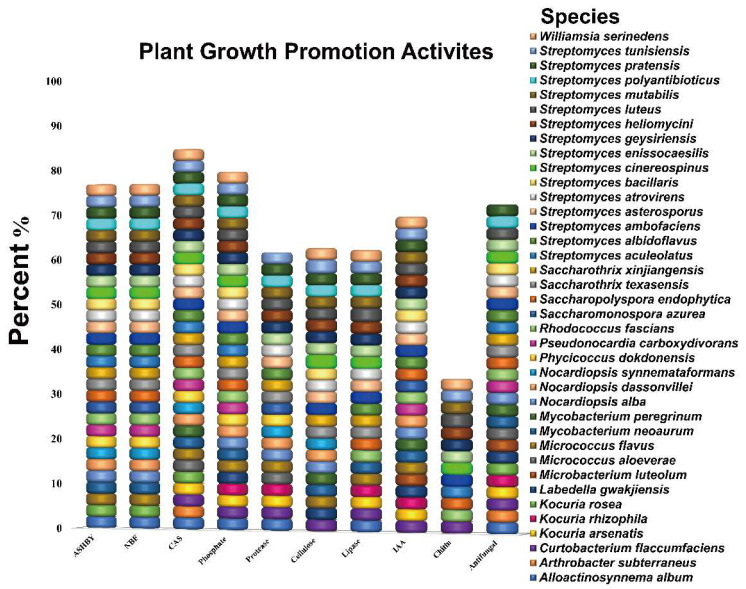
Plant growth-promotion activities of endophytic actinobacterial strains from medicinal plant *Thymus roseus* in vitro. All experiments were performed twice with three replicates for each individual strain.

In the present study, 71 strains were screened for multiple beneficial traits in vitro to determine the promising endophytic actinobacterial strains (Figure S1). Data obtained in this study revealed that the majority of tested strains exhibited one or more plant growth-promoting activities. In our screening findings, the highest percent of tested strains were able to produce siderophores (84%) followed by phosphate solubilization (79%), and the majority of these strains belonged to *Streptomyces*, *Mycobacterium*, *Nocardiopsis*, *Saccharopolyspora*, *Micrococcus*, *Curtobacterium*, *Alloactinosynne ma*, *Williamsia*, *and Pseudonocardia* genera. Similarly, most of the tested strains showed nitrogenase activity (76%) based on growth on both NFC and Ashby’s free nitrogen medium, including *Streptomyces*, *Saccharothrix*, *Saccharopolyspora*, *Nocardiopsis*, *Alloactinosynnema*, *Pseudonocardia*, *and Mycobacterium* genera (Figure 1). Among all tested strains, about 69% were able to synthesize IAA, including members of the genera *Williamsia*, *Streptomyces*, *Saccharopolyspora*, *Rhodococcus*, *Pseudonocardia*, *Nocardiopsis*, *Mycobacterium*, *Micrococcus*, *and Microbacterium* genera (Figure 1). Additionally, most of the endophytic actinobacterial strains produced one or more hydrolytic enzymes: protease (61%), cellulose (64%), lipase (62%), and chitinase (34%) (Figure 1).

Furthermore, all endophytic actinobacterial strains were individually tested against the fungal pathogen *Verticillium dahliae* Kleb, which causes cotton wilt diseases in vitro. Of 71 strains examined, (71%) displayed inhibitory activity with the percentage of inhibition ranging from 12.5 to 42.0% against *Verticillium dahliae* Kleb in vitro (Figure 1). As evidenced in preliminary tests of our assays, several endophytic bacteria displayed antagonistic effects against *V. dahliae*. The majority of tested strains were representing the *Streptomyces* genus, followed by *Mycobacterium*. Based on our results, the largest inhibition zone (42.0%) was observed for strain XIEG24, which belonged to *Streptomyces albidoflavus,* followed by *Nocardiopsis alba* XIEG57 (30.3%), and *Curtobacterium flaccumfaciens* XIEG24 (29.1%) (Supplementary Table S1). In light of the screening results, strains XIEG24, XIEG29, and XIEG57 were selected for their high antagonistic activity to *V. dahliae* in subsequent pot experiments under salt stress.

### 3.2. Stimulation of Cotton Growth by Endophytes under Salt Stress in a Pot Experiment 

To explore the plant growth-promoting (PGP) effects in conditions of salt stress. Three strains, *Streptomyces luteus* (XIEG05), *Nocardiopsis dassonvillei* (XIEG12), and *Alloactinosynnema album* (XIEG20) were positive for at least six plant-beneficial traits in vitro including IAA production, phosphate solubilization, nitrogenase activity, siderophore, and at least production of two hydrolytic enzymes were selected to test their plant growth stimulation properties in pot experiments with cotton plants under salt stress (Supplementary Table S1).

**Figure 2:**
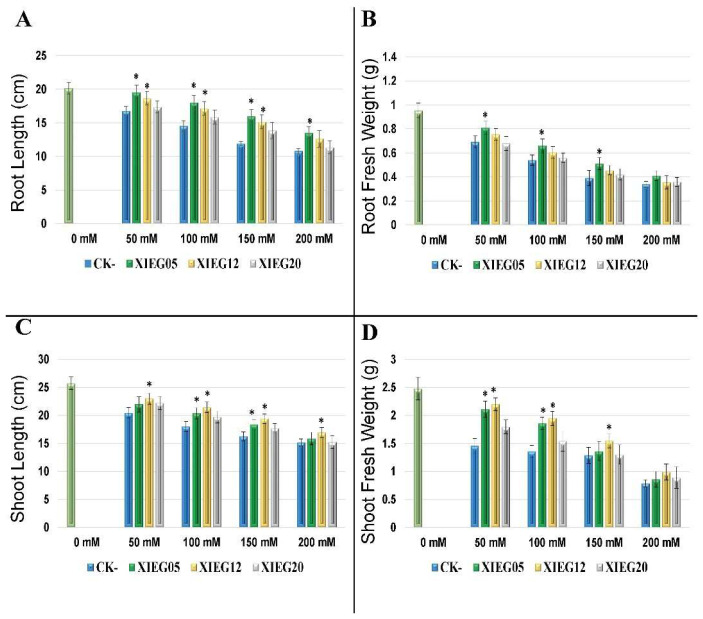
The response of cotton 60 days after inoculation with the selected endophytic actinobacteria under salt stress, compared with an uninoculated control plant. In this experiment, the data represents a mean of at least 10–12 replicates ± standard error (SE). The column marked by (“*”) indicate significant differences based on One-way ANOVA, followed by Tukey’s HSD post-hoc test for multiple comparisons at alpha level = 0.05. (**A**) Root length; (**B**) Root fresh weight; (**C**) Shoot length; (**D**) Shoot fresh weight.

Our results in this pilot study showed that cotton growth under different NaCl concentrations was strongly impaired by salinity when cotton was not inoculated with selected endophytic actinobacterial strains, evidenced by decreased root and shoot length and total fresh weight compared to non-stressed control (Figure 2). On the other hand, in comparison with uninoculated cotton under salt stress, the supplementation of strains XIEG05 and XIEG12 significantly (*p* < 0.05) increased cotton root and shoot length and total fresh weight at different salt concentrations, while strain XIEG20 showed slightly increased, compared to the uninoculated controls. In addition, notably that these results indicated that all tested strains in this study have a different response in terms of cotton growth at certain salt concentrations.

For instance, inoculation with strain XIEG05 showed the strongest stimulation of the cotton root length significantly (*p* < 0.05) by 16.5, 23.9, 34.4, and 25.1% at 50, 100, 150, and 200 mM NaCl, respectively, compared to the uninoculated controls (Figure 2A). In addition, strain XIEG05 increased the fresh weight of root by 17.4, 22.3, 30.8, 20.6% at 50, 100, 150, and 200 mM NaCl, respectively, compared to the uninoculated controls (Figure 2B). For shoot length, co-cultivation with strain XIEG12 showed the strongest stimulatory effect on cotton growth was obtained at 50, 100, 150, and 200 mM by 12.5, 18.9, 19.5, and 11.7%, respectively, compared to the uninoculated controls (Figure 2C). For shoot fresh weight, strain XIEG12 showed the highest increase significantly (*p* < 0.05) of the growth rate by 50.9, 43.3, 20.5, and 25.8% at 50, 100, 150, and 200 mM, respectively, compared to the uninoculated control (Figure 2D). Overall, our results showed that strains XIEG05, XIEG12, and XIEG20 enhanced plant tolerance to salt stress through the promotion of shoot and root development.

### 3.3. Determination of Antioxidant Enzyme Activity

**Figure 3:**
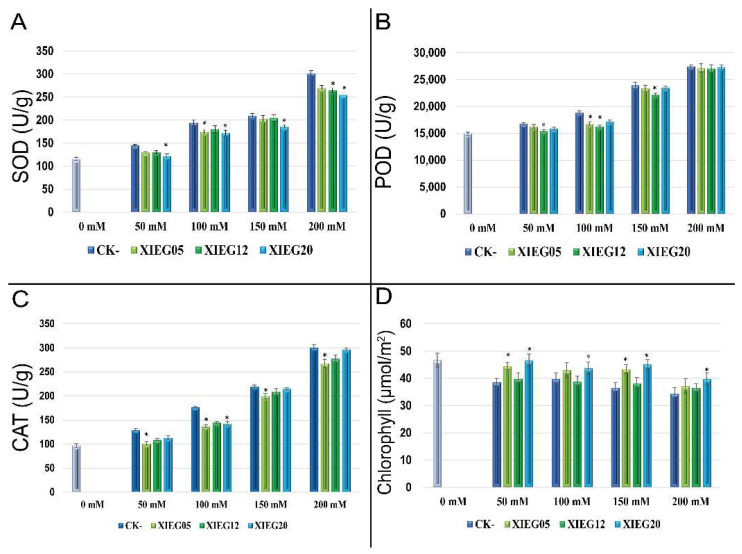
Activities of antioxidant enzymes under salt stress in the presence and absence of selected endophytic actinobacterial strains from *Thymus roseus*. (**A**) Superoxide dismutase (SOD); (**B**) peroxidase (POD); (**C**) catalase (CAT); and (**D**) photosynthetic pigments. This experiment was conducted twice in triplicate and the mean of at least 3 replicates ± standard error (SE) was calculated for SOD, POD, and CAT. But for photosynthetic pigments (*n* = 12). The column marked by (“*”) indicate significant differences based on One-way ANOVA, followed by Tukey’s HSD post-different test for multiple comparisons at alpha level = 0.05.

Salt stress induced production provokes oxidative stress (ROS) like superoxide dismutase (SOD), catalase (CAT), and peroxidase (POD). To minimize the deleterious effects of ROS, we investigated the effect of three endophytic actinobacterial strains, *Streptomyces luteus* (XIEG05), *Nocardiopsis dassonvillei* (XIEG12), and *Alloactinosynnema album* (XIEG20), which showed plant growth stimulation under saline conditions (50–200 mM NaCl) on cotton plant physiological parameters involved in defense systems against oxidative stress to cope with salinity stress. The results pertaining to the effects and alleviating of salt stress on antioxidant enzymes activities by all tested strains are reported in (Figure 3).

The superoxide dismutase (SOD) activity increased with increasing salinity levels. The highest SOD concentration observed under the salinity level at 50, 100, 150, and 200 mM NaCl was 36.6, 45.4, 49.4, and 64.8%, respectively, as compared to non-stressed control (Figure 3A). However, the application of strain XIEG20 showed the highest decrease significantly (*p* < 0.05) of SOD enzyme by 15.7, 11.5, 12.4, and 15.6%, at 50, 100, 150, and 200 mM NaCl, respectively, compared with salt treatments without application of endophytic actinobacterial strains (Figure 3A).

In the leaves of cotton, the activity of POD was positively and progressively correlated with the salinity level. POD content increased by 12.0, 25.9, 60.0, and 83.3% at 50, 100, 150, and 200 mM NaCl, respectively, as compared to non-stressed control (Figure 3B). Moreover, it is clear that the supplementation of strain XIEG12 significantly (*p* < 0.05) reduced the peroxidase activity at 50, 100, and 150 mM NaCl by 8.5, 16, 7.8%, respectively, in comparison to the uninoculated controls. However, this effect was not significant at 200 mM NaCl.

The level of CAT activity gradually increased during all stages of the salt stress treatments. In particular, the results showed that the CAT activity increased due to salinity stress by 33.5, 82.5, 126.3, and 210% at 50, 100, 150, and 200 mM NaCl treatments, respectively, as compared to non-stressed controls (Figure 3C). Whereas CAT activity was reduced considerably in the inoculated plants by tested endophytic actinobacterial strains and the highest reduction was observed by strain XIEG05 at 50, 100, 150, and 200 mM NaCl reached 28.1, 28.7, 9.7, and 12.0%, respectively, when compared to that of the untreated control (Figure 3C).

The results pertaining to the effect of salt stress on chlorophyll contents in the presence and absence of tested endophytic actinobacterial strains are shown in (Figure 3D). Our results showed that cotton exhibited considerable reductions in chlorophyll under salt stress conditions with increased salt stress levels by 18.4, 14.8, 24.8, and 32.7% at 50, 100, 150, and 200 mM NaCl, respectively, as compared to non-stressed controls (Figure 3D). Conversely, at different salinity levels, the inoculation of cotton with all tested endophytic actinobacterial strains has resulted in a drastically increased in chlorophyll synthesis. The results reveal that the application of strain XIEG20 recorded the highest increased significantly (*p* < 0.05) in chlorophyll at 50, 100, and 150 mM NaCl by 15.3, 8.2, and 18.7% at 50, 100, and 150 mM NaCl, respectively, while a slight increase was observed in 200 mM NaCl treatment in comparison to the uninoculated controls. In the present study, the data presented in Figure 3 indicated that the activities of antioxidant enzymes (SOD, POD, and CAT) were positively and progressively increased correlated with the salinity level while the reverse was true for the co-inoculation of cotton with tested endophytic actinobacterial strains as compared with NaCl treatments. 

### 3.4. Biological Control of V. dahliae In Vivo under Salt Stress

**Figure 4:**
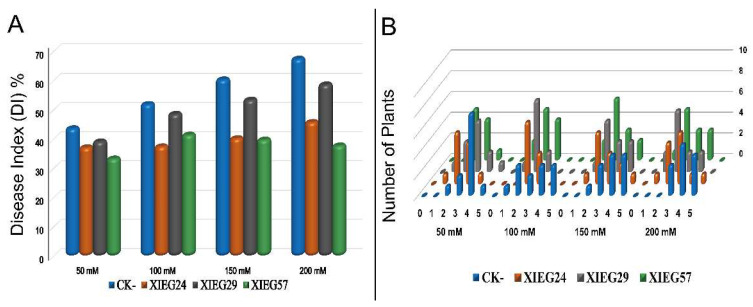
Effect of endophytic actinobacterial strains on disease index and disease grades of cotton plants to *Verticillium dahliae* over eleven weeks under salt stress. (**A**) Disease index of cotton plants with and without endophytes under salt stress. (**B**) Signs of disease grades rated from ‘0’ to ‘4’ 0 (no symptoms), 1 (>0–25% yellowing or wilting leaves), 2 (25–50% yellowing or wilting leaves), 3 (50–75% yellowing or wilting leaves), and 4 (75–100% yellowing or wilting leaves).

To explore the biological control effect of three endophytic actinobacterial strains named *Streptomyces albidoflavus* (XIEG24), *Curtobacterium flaccumfaciens* (XIEG29), and *Nocardiopsis alba* (XIEG57) were chosen for a pot experiment under greenhouse conditions after evaluating their ability for antagonistic activity in vitro to test their ability to suppress cotton disease caused by *V. dahliae* under salt stress in vivo. About 12 days after fungal inoculation, the first signs of aerial wilt symptoms developed on Verticillium-inoculated cotton appeared as blotchy, consisting of chlorotic areas between the main veins of the lower leaves on the first and second mature leaves, and twig dieback that ultimately browned of leaves and defoliated of the non-inoculated cotton at different salt concentrations. The application of *V. dahliae* on cotton (uninoculated control) increased the disease severity index (DSI) up to 43.7, 52.0, 60.4, and 67.5% at 50, 100, 150, and 200 mM NaCl, respectively, as compared to the uninoculated controls (Figure 4A).

After 8 weeks, our results showed that cotton seedling bacterization with three antagonistic endophytic actinobacterial strains XIEG24, XIEG29, and XIEG57 reduced the cotton disease incidence symptoms caused by *V. dahliae* compared to non-bacterized control. Noticeably, the results presented in (Figure 4A) indicated that cotton seedling bacterization with three selected antagonistic strains could slow disease development, and the expression of signs was delayed compared to the control plantlets under pathogen-challenged at different salt concentrations. For instance, our potential biocontrol strain XIEG57 exhibited the highest reduction of cotton disease incidence (DI) symptoms caused by *V. dahliae* up to 33.3, 37.5, 35.3, and 37.8% at 50, 100, 150, and 200 mM NaCl, respectively, compared to uninoculated plants. Although all tested endophytic actinobacterial strains conferred some degree of Verticillium wilt resistance, the distribution of disease grades varied dramatically at different salt concentrations compared to uninoculated controls (Figure 4B).

### 3.5. Identification of Bioactive Compound by Gas-Chromatography/Mass-Spectrometry (GC-MS)

**Figure 5:**
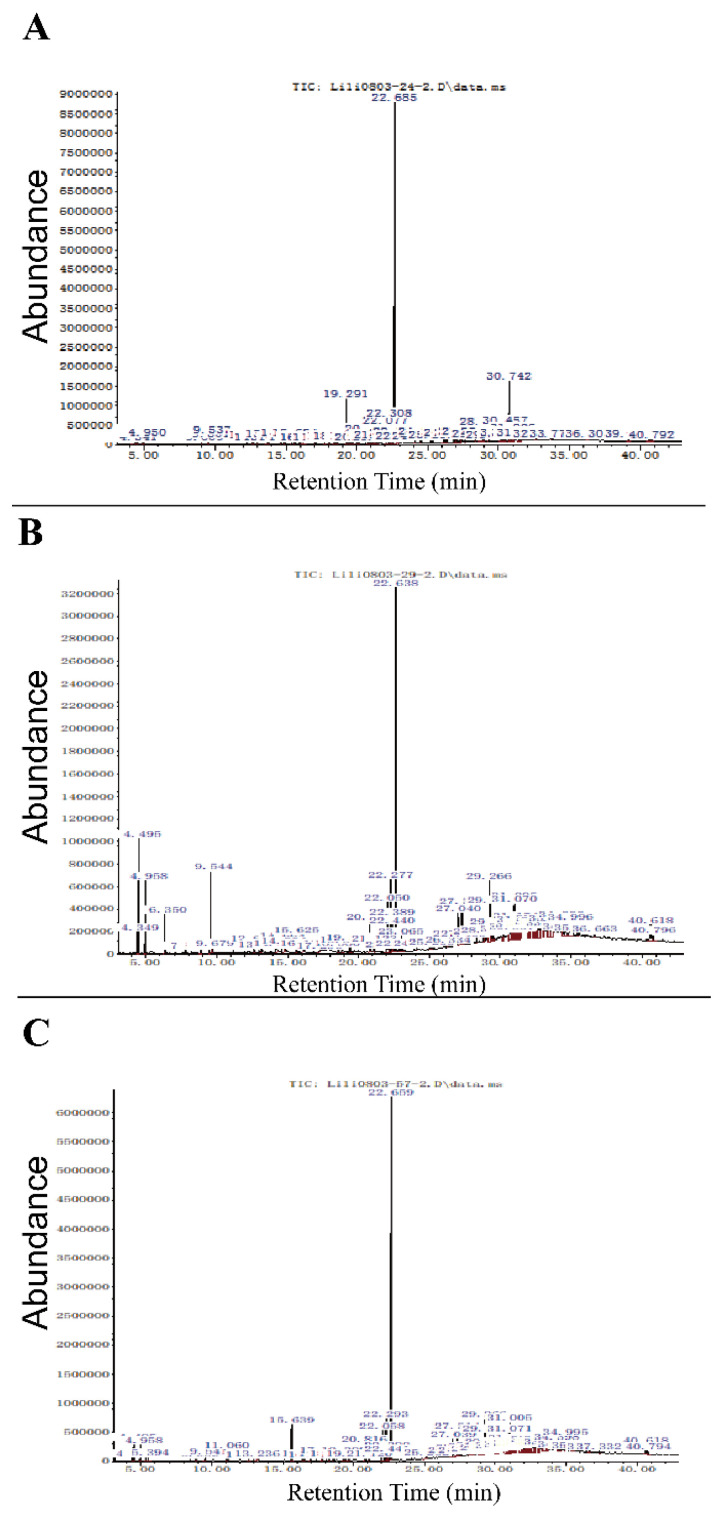
GC-MS analysis of potential bioactive compounds in ethyl acetate extracts of cell supernatant buffered of four co-cultures endophytic actinobacterial strains, (**A**) *Streptomyces albidoflavus* (XIEG24), (**B**) *Curtobacterium flaccumfaciens* (XIEG29), and (**C**) *Nocardiopsis alba* (XIEG57) with *V. dahliae*.

Ethyl acetate extracts of cell supernatant buffered of three a co-culture endophytic actinobacterial strains, *Streptomyces albidoflavus* (XIEG24), *Curtobacterium flaccumfaciens* (XIEG29), and *Nocardiopsis alba* (XIEG57) with *V. dahliae* were studied by GC-MS (Figure 5). Each peak represents a discrete chemical compound and the interpretation on mass spectrum GC-MS was conducted using the database of the National Institute Standard and Technology (NIST).

The GC-MS analysis of crude extracts buffered from strain *Streptomyces albidoflavus* (XIEG24) with *V. dahliae* showed 72 peaks (Figure 5A). The mass spectrum of strain XIEG24 showed that there were four major compounds in ethyl acetate extracts suggestive of Benzene, 1,2,4-triethyl-, Dibutyl phthalate, Bicyclo [3.1.1] heptan-2-one, and 6,6-dimethyl- (Supplementary Table S2). For strain *Curtobacterium flaccumfaciens* (XIEG29) with *V. dahliae*, the GC-MS resolved several features in the extracts: 72 compounds in total (Figure 5B), 15 of them were characterized by mass analyzer detector GC/MS as high peaks including p-Xylene, Benzene, 1,3-dimethyl-, Benzaldehyde, Phenylethyl Alcohol, Phenol, 3,5-dimethoxy-, l-Proline, N-allyloxycarbonyl-, Diethyltrisulphide, l-Proline, N-allyloxycarbonyl-, Dibutyl phthalate, 9-Octadecenamide, (Z)-, Pyrrolo [1,2-a]pyrazine-1,4-dione,1-Docosene, Cyclooctacosane, and Nonacos-1-ene (Supplementary Table S3). 

About 81 compounds were identified by GC-MS for strain *Nocardiopsis alba* (XIEG57) with *V. dahliae*, (Figure 5C), the mass spectrum showed that 10 major peaks were obtained from ethyl acetate extracts suggestive of Dimethyl phthalate, l-Leucine, N-cyclopropylcarbonyl, Pyrrolo [1,2-a] pyrazine-1,4-dione, Dibutyl phthalate, 1-Docosene, Triacontyl acetate, 13-Docosenamide, (Z)-, Nonacos-1-ene, and 1-Hexacosene (Supplementary Table S4). Moreover, several minor peaks were present in the crude extracts of four co-culture endophytic actinobacterial strains XIEG24, XIEG29, and XIEG57 are presented in (Tables S2–S4). In the present study, most of the compounds revealed by GC-MS were mainly fatty acid esters, phenols, alkanes, alkenes, and aromatic chemicals.

## 4. Discussion

Salinity stress is one of the major abiotic stress suppressions limiting crop production in arid and semi-arid regions throughout the world. It has been reported that among the various microbial inoculants, endophytic microorganisms are the most promising beneficial microorganisms for their beneficial exploitation in the field of agriculture [18,63]. In particular, endophytic microorganisms can play a significant role as eco-friendly biofertilizers and biocontrol agents for crop disease management [48,50,53]. However, despite the previous work done earlier, there is still limited knowledge on the role of endophytic actinobacteria from different medicinal plants with potential plant growth promotion and antifungal activity [20,46,64]. Therefore, understanding the mechanism underlying plant response to salinity provides new insights into the improvement of salt tolerance crops.

In the present investigation, it has also been documented that endophytic actinobacterial strains exhibited multiple plant beneficial activities, such as the production of IAA (69%), siderophores (84%), phosphate solubilization (79%), and nitrogenase activity (76%). The findings of our study are parallel to some of the previous investigations which reported that endophytes produced several plants promoting traits including the production of phytohormones, siderophores, phosphate solubilization, nitrogenase activity, and cell-wall-degrading enzymes [48,65,66,67,68,69,70]. In addition, many of the endophytic actinobacterial strains screened in this study were able to produce at least one of the hydrolytic enzymes. The production of cell-wall hydrolyzing enzymes such as cellulose (64%), protease (61%), lipase (62%), and chitinase (34%) are involved in minimizing the challenges imposed by phytopathogens and may play an important role in the biocontrol of plant diseases which have been well-documented previously by various researchers [26,48,53,71,72,73].

Based on the obtained results, the growth of cotton under salt stress was strongly impaired with increasing salinity when cotton was not co-inoculated with selected endophytic actinobacterial strains. In this regard, in response to salinity stress, the maximum decrease recorded in the root, shoot length, and root, shoot weight of cotton were 32.1, 31.5, 29.5, and 33.3%, respectively, at all salt concentrations (Figure 2). Contrarily, at different salinity levels, the application of selected beneficial plant-microbe *Streptomyces atrovirens* XIEG05 showed the strongest stimulation of root length and root weight as compared to the controls. Likewise, the highest increase in the shoot length and shoot weight was obtained by inoculation of *Alloactinosynnema album* strain XIEG12, compared to the controls. Accordingly, we observed that the selected salt-tolerant endophytic actinobacterial strains improved the symbiotic performance of cotton under saline conditions. Data obtained in Figure 2 showed that all selected endophytic actinobacteria in this investigation demonstrated a significant role in plant growth activity and displayed increased physiological parameters such as plant biomass and plant length at different salt concentrations. The obtained results are in harmony with many previous studies which suggested that the application of endophytic actinobacteria can increase the growth of different crops such as wheat, rice, lettuce, tomato, pepper, canola, and bean during exposure to salt stress [74,75,76,77,78,79,80,81,82,83,84,85].

Our study showed that the co-cultivation with strain XIEG05 and XIEG12 enhanced cotton growth and salinity tolerance, especially in the development of primary root length, by regulating cell division and differentiation. Apparently, all tested strains appear to influence the root system architecture via root developmental changes, which might enhance plant tolerance to salt stress by the stimulated root system and could explain the enhanced capacity of the plant by solubilizing mineral nutrients and facilitating their availability to plants to acquire and utilize more nutrients which are in agreement with the findings of [86,87,88]. On the other hand, our data recorded that the co-inoculation of XIEG05, XIEG12, and XIEG20 strains induced the accumulation of antioxidant compounds to reduce the impact of salt stress as compared to uninoculated plants (Figure 3), which is in agreement with the findings of [24,48,89]. For instance, inoculation cotton with strain XIEG05 reduced CAT activity at different salt concentrations compared to uninoculated controls (Figure 3C); a similar result was observed by [90,91]. Moreover, our observations demonstrated that strain XIEG20 decreased the activity of SOD significantly as compared to the uninoculated controls (Figure 3A). Kadmiri et al. [92] found in their study that the inoculation of *Pseudomonas fluorescens* Ms-01 on wheat grown under salt stress decreased the activity of SOD, which improves the defense pathway. In the present experiment, the highest chlorophyll pigments were observed at all salt concentrations via application of strain XIEG20 as compared to uninoculated plants (Figure 3D). Our results are in accordance with the findings of Inderbitzin et al. [93], who reported the effects of water stress and inoculation with beneficial microorganisms on antioxidant status and photosynthetic pigments in basil (*Ocimum basilicum* L.).

Controlling Verticillium wilt is challenging since the pathogen *V. dahliae* can survive in the soil for a long time in dormant forms and causes wilt diseases and crop losses of varying severity, as well as natural ecosystems [94]. The signs of *Verticillium* wilt disease start with yellowing, followed by chlorosis and necrosis of leaves [53]. In the present investigation, in non-inoculated cotton, salt stress increased the portion of diseased plants up to 43.7, 52.0, 60.4, and 67.5% at 50, 100, 150, and 200 NaCl, respectively, whereas in the presence of strains *Streptomyces albidoflavus* (XIEG24), *Curtobacterium flaccumfaciens* (XIEG29), and *Nocardiopsis alba* (XIEG57) the portion of plants that exhibited disease symptoms decreased at a different level of salt treatments (Figure 4). These observations demonstrated that the ability of endophytes to colonize internal plant tissues and protect plants from soilborne pathogens was reviewed by [95]. However, our potential biocontrol strain XIEG57 exhibited the highest disease reduction compared to uninoculated controls. The findings from our study are in agreement with recent reports of [96,97,98], which stated that *Nocardiopsis.* sp produces different volatile organic compounds (VOCs) as a biofungicide. Moreover, endophytic actinobacteria associated with medicine plants may play an important role in suppressing the growth of fungal pathogens and can minimize the challenges imposed by phytopathogens including the genus Streptomyces, which are well known for their ability to synthesize different secondary metabolites that play an important role in the biological control of plant disease [18,20,26,64,72,99].

To further understand the underlying mechanism, we conducted Gas-Chromatography/Mass-Spectrometry (GC-MS) analysis to identify the antimicrobial compounds produced by antagonistic strains. Although it is hard to judge whether the components identified by GC-MS are from bacteria or fungi; however, the analysis of obtained data revealed that co-cultures of strains XIEG24, XIEG29, and XIEG57, with *V. dahliae*, were tentatively identified as compounds with known antimicrobial, antiphrastic, antitumor, and anticancer properties. Data obtained in (Figure 5A) showed that the major peaks in cell-free extracts from the co-culture of strain *Streptomyces albidoflavus* (XIEG24) are known as antimicrobial compounds such as Dibutyl phthalate [100], Bicyclo [3.1.1] heptan-2-one [101].

For strain *Curtobacterium flaccumfaciens* (XIEG29), the GC-MS resolved several antimicrobials compounds in the extracts as major peaks such as p-Xylene, Benzene, 1,3-dimethyl-, Benzaldehyde [102], Phenylethyl Alcohol [103], and also Phenol, 3,5-dimethoxy-, which is well-known as a polyphenolic compound with antifungal and antibacterial activities [104]; Diethyl trisulphide [105], 9-Octadecenamide [106], Pyrrolo [1,2-a] pyrazine-1,4-dione [107], 1-Docosene and 1-nonadecene are alkenes [108], and Nonacos-1-ene [109] (Figure 5B).

In this study, about 81 compounds were identified by GC-MS for strain *Nocardiopsis alba* (XIEG57); the major peaks in (Figure 5D) showed antibacterial activity such as Dibutyl phthalate [100], 1-Docosene, and 1-nonadecene [108], 1-hexadecene [110], and antifungal activity against *Pyricularia oryzae* such as Pyrrolo [1,2-a] pyrazine-1,4-dione, hexahydro-3-(2-methylpropyl)-, and Nonacos-1-ene [109], and 13-Docosenamide, (Z)- [110]. Overall, our results supported the development of natural products that may minimize the need for the application of chemical fertilizer and fungicides, which would be an environmentally friendly approach and preserve biological resources in a sustainable agricultural system.

## 5. Conclusions

Our study revealed that endophytic actinobacterial associated with medicinal plant *T. roseus* produced multi-plant growth-promoting substances including IAA, solubilized phosphate, fixed nitrogen, production of siderophores, and produced lytic enzymes (i.e., lipase, cellulase, protease, and chitinase) in vitro. Overall, the greenhouse experiments in this study showed that in response to high salinity stress, inoculation of the selected endophytic actinobacterial strains *Streptomyces luteus* (XIEG05) and *Nocardiopsis dassonvillei* (XIEG12) were significantly (*p* < 0.05) enhanced cotton growth promotion at different salinity levels (50–200) mM compared to un-inoculated cotton. In addition, each tested strain in this study had a different response in terms of cotton growth at certain salt concentrations. In the present study, the activities of antioxidant enzymes (SOD, POD, and CAT) were positively and progressively increased correlated with the salinity level, while the reverse was true for the co-inoculation of cotton with tested endophytic actinobacterial strains as compared with NaCl treatments. 

Moreover, the inoculation of cotton with tested endophytic actinobacterial strains resulted in a drastic increase of the chlorophyll synthesis. Noticeably, the results indicated that the three selected antagonistic endophytic actinobacterial strains *Streptomyces albidoflavus* (XIEG24), *Curtobacterium flaccumfaciens* (XIEG29), and *Nocardiopsis alba* (XIEG57) could slow disease development, and the expression of signs was delayed compared to the control plantlets under pathogen-challenged at different salt concentrations. Although all tested endophytic actinobacterial strains conferred some degree of *Verticillium* wilt resistance, the distribution of disease grades varied dramatically at different salt concentrations compared to uninoculated controls. In addition, most compounds revealed by GC-MS were mainly fatty acid esters, phenols, alkanes, alkenes, and aromatic chemicals and have been reported to have antibacterial and antifungal activity. 

In summary, our results provide insights about plant beneficial traits of endophytic bacteria associated with the medicinal plant *T. roseus* and their interactions with cotton under stressful environments. Moreover, these results support the development of natural products that may minimize the need for the application of chemical fertilizer and fungicides, which would be an environmentally friendly approach and preserve biological resources in a sustainable agricultural system. Further research is needed for future applications in cotton plant growth promotion and crop productivity, as well as to verify the biological control efficacy of selected endophytic bacteria against *V. dahliae* in the field experiment.

## Data Availability

Raw sequence data reported in this paper have been deposited in the GenBank in the NCBI under accession numbers (MN686679–MN686702(24), MN687832–MN687853(22), MN688237–MN688255(19), MN688672–MN688674(3), MN688677(1), MN688679–MN688680(2)).

## Supplementary Materials

The following supporting information can be downloaded at: https://zenodo.org/record/6361855#.Ylzt0uhBzGJ (accessed on 16 March 2022), Figure S1: Confirmation of plant growth promotion traits by a color change or halo zone on the selective medium; Figure S2: Effect of endophytic actinobacterial strains on the growth of cotton under salt stress compared with an uninoculated control (CK−); Table S1: The beneficial traits in selected endophytic bacteria from *Thymus roseus* in vitro; Table S2: GC-MS identified components of the antibiosis crude extract of XIEG24 and V. dahliae mixture at pH7. Volatile Compounds are listed in ascending order of Retention with at least percentage match ≤70%; Table S3: Supplementary Table S3: GC-MS identified components of the crude extract of XIEG29 at pH7. Volatile compounds are listed in ascending order of Retention Time with at least percentage match ≤70%); Table S4: GC-MS identified components of the antibiosis crude extract of XIEG57 and V. dahliae mixture at pH7. Volatile Compounds are listed in ascending order of Retention Time with at least percentage match ≤70%.
